# Disruption of the preB Cell Receptor Complex Leads to Decreased Bone Mass

**DOI:** 10.3389/fimmu.2019.02063

**Published:** 2019-09-04

**Authors:** Mohamed Khass, Harunur Rashid, Peter D. Burrows, S. Louis Bridges, Amjad Javed, Harry W. Schroeder

**Affiliations:** ^1^Department of Medicine, University of Alabama at Birmingham, Birmingham, AL, United States; ^2^Genetic Engineering and Biotechnology Division, National Research Center, Cairo, Egypt; ^3^Department of Oral and Maxillofacial Surgery, University of Alabama at Birmingham, Birmingham, AL, United States; ^4^Department of Microbiology, University of Alabama at Birmingham, Birmingham, AL, United States

**Keywords:** preB cell receptor, surrogate light chain, bone development, B cell signaling, adult bone mass

## Abstract

In the bone marrow, preB cells are found adjacent to the bone endosteum where bone synthesizing osteoblast and bone resorbing osteoclasts reside. Although there is evidence of interactions between preB and bone cells, the factors that contribute to such interactions are poorly understood. A critical checkpoint for preB cell development assesses the integrity of the nascent immunoglobulin μ heavy chain (HC) by testing whether it can participate in the formation of a preB cell receptor (preBCR), composed of the μ HC and surrogate light chain (LC). In this work, we tested whether loss of preBCR components can affect bone synthesis. A panel of gene targeted mice with sequential blocks in preBCR formation or function [surrogate light chain component lambda 5 deleted (λ5^−/−^), transmembrane domain of μHC deleted (IgM-mem^−/−^), and CD19 preBCR co-receptor deleted (CD19^−/−^)] were evaluated for effects on postnatal bone synthesis. Postnatal bone mass was analyzed in 6 month old mice using μ-CT, histomorphometry and double calcein labeling. Both cortical and trabecular bone mass were significantly decreased in the femurs of the λ5 and IgM-mem deficient mice. Histomorphometric analysis showed a decrease in the numbers of osteoblasts and osteoclasts in all three mutant strains. Double calcein labeling revealed a significant decrease in dynamic synthesis and mineralization of bone in λ5^−/−^ mice. Our data strongly suggest that interference with preBCR formation or function affects bone homeostasis independent of the presence or absence of mature B cells, and that components of the preBCR play important, and potentially distinct, roles in regulating adult bone mass.

## Introduction

In the bone marrow, B cell progenitors are found adjacent to the endosteum where osteoblasts, osteoclasts, and other bone elements reside (1). Defects in bone formation have been shown to impair preB cell development (2) and conversely, factors influencing B cell development have been shown to affect bone. For example, in osteoclast impaired osteopetrotic mice the transition of proB to preB cell is obstructed (3). PreB cell development is blocked in osteopetrotic RANK or RANKL deficient mice, as well as in mice where osteoblasts are inhibited or overstimulated (4, 5). Osteoporotic OPG-null mice have increased numbers of preB cells (6). Conversely, IL-7R deficient mice, which exhibit a block at the preB cell stage, show increased bone mineral density (7). Overexpression of IL-7 leads to both increased preB cell numbers and increased bone resorption (8). Deficiency of Pax-5, a key transcription factor for preBCR expression, leads to a block at the proB cell stage (9) and increased numbers of osteoclasts (4, 10, 11). Loss of early B cell factor, a transcription factor that controls preBCR expression (12), leads to a decreased osteoclast numbers and an increase in osteoblast numbers and bone formation (13).

The preB cell receptor (preBCR) plays a key role in controlling the early development of preB cells, which are positioned to potentially influence the cells involved in bone formation and remodeling. There are also parallels in their function. For example, aged individuals demonstrate both decreased expression of surrogate light chain (SLC) (14) and decreased functional activity and numbers of osteoblasts (15, 16). Mice with mutations in Bruton's tyrosine kinase, which affects preBCR signaling, exhibit impaired osteoclast maturation (17).

To test the role of preBCR in maintenance of adult bone mass, we used a panel of mouse models where three components of the preBCR signaling complex—CD19, the transmembrane domain of the μHC (IgM-mem), and λ5—have undergone individual loss of function mutations. We used this panel of mutant and wild-type (WT) mice to test the role of CD19 co-receptor function, membrane bound heavy chain, and SLC in adult bone mass. We found that each of these preBCR factors has a separate effect on total bone mass in 6 month-old mice.

## Materials and Methods

### Mouse Models

The generation of CD19^−/−^, IgM-mem^−/−^, and λ5^−/−^ mice was previously described (18–20). Genotypes were verified by PCR using DNA from a tail biopsy with Direct PCR lysis reagent (Viagen Biotech; Los Angeles, CA). The targeted alleles were confirmed by using gene-specific primer pairs and by evaluation of B cell populations by flow cytometry. The deletion of λ5 was confirmed by using the forward primer 5′GGAGATCTACACTGCAAGTGAGGCT3′ and reverse primer 5′ACACTGGCCTTGCAATTGATCGAG 3′.

All mouse lines were maintained on a C57BL/6 background. Only female mice were used in this study. All mice were housed under a 12 h light:dark cycle with *ad libitum* access to food and water. Limbs were harvested from 6 month old mice for assessment of bone quality. All animal experiments were performed with the approval from the Institutional Animal Care and Use Committee of the University of Alabama at Birmingham and conformed to relevant federal and state guidelines and regulations.

### Histological Analysis and Double Calcein Labeling

Hind limbs from 6 month old wild-type and mutant mice were harvested and fixed in 4% paraformaldehyde in PBS overnight at 4°C. Fixed tissues were dehydrated through an ethanol gradient, cleared in xylene, and embedded in Methyl Methacrylate (MMA). Embedded femurs were sectioned laterally at 5 μm thickness and mounted on Superfrost Plus slides (Fisher Scientific). Serial sections were then stained with Goldner's Trichrome or TRAP as described previously (21). Sections were analyzed by digital microscopy and images were collected with a Nikon Eclipse 80i color camera.

To assess osteoclast activity, femur sections were TRAP stained. The number and surface area of multinucleated osteoclasts were then calculated as per ASBMR guidelines (22). The erosion surface and quiescent surface on the TRAP stained sections were quantified using The Bioquant Osteo v18.2.60 software (BIOQUANT Image Analysis Corporation, Nashville, TN) and according to ASBMR guidelines (22). Erosion surface represents the total percentage of bone surface that shows signs of resorption (a crenated or lacunar surface underneath the TRAP positive osteoclast surface). Quiescent surface represents resting or inactive surface of bone that is devoid of cuboidal osteoblasts or TRAP positive osteoclasts.

Double calcein labeling was performed to evaluate dynamic mineral apposition and bone formation. Six month old mice received an intraperitoneal injection of 20 mg of calcein (Sigma-Aldrich) per kg of body weight in a 2% sodium bicarbonate solution. Five days later, mice received a second injection of calcein. Limbs were collected 2 days later and processed for histologic sectioning and histomorphometry.

To determine the calcein label, unstained femur sections were viewed under fluorescent light. Analysis of bone formation rate (BFR) was performed using The Bioquant Osteo v18.2.60 software (BIOQUANT Image Analysis Corporation, Nashville, TN). The length of the single labeled surfaces and double labeled surfaces was measured by tracing the Calcein signal along the surface and the width between the two labelled surfaces was measured at many intervals and then averaged. The average width along with the labeling period (days between injections) was then used to calculate the mineral apposition rate (MAR) and the BFR. BFR is the volume of mineralized bone formed per unit time and per unit bone surface.

### Histomorphometry and μ-CT Analysis

For histomorphometric analysis, 5 μm lateral sections of undecalcified femur were processed as described above and stained with Goldner's trichrome. Analyses on lateral sections midway through the femur were performed using the Bioquant Osteo semi-automated system for skeletal phenotyping. Nomenclature, symbols, and units used are those recommended by the Nomenclature Committee of the American Society for Bone and Mineral Research (22). All histomorphometric analyses were performed on three independent sections and assessed by blinded examiners.

To perform histomorphometric analysis of osteoblast and osteoclast parameters we stained sections of femurs using Mason's trichrome then imaged by microscope. For quantification of trabecular bone, a 250 μm region beneath the growth plate and a depth of 1 mm off of the peak of the growth plate region is selected as region of interest. All of the trabecular bone (stained blue) in the region is traced and measured for volume and bone surface. The Bioquant Osteo v18.2.60 software (BIOQUANT Image Analysis Corporation, Nashville, TN) takes the measurements and then assigned the bone surface as either osteoblast surface (cuboidal osteoblast at the bone surface) or osteoclast/erosion surface (TRAP stained). The number of osteoblast (cuboidal cells at the bone surface) and osteoclast (multinucleated TRAP positive cells) are then manually counted from the images of same area captured at high magnification. Enumeration is performed by three blinded evaluators and the data is averaged to minimize personal bias. The average data is presented in the graph in our manuscript.

Three dimensional bone structure and mineral density were assessed by micro-computed tomography. Femurs were dissected from female mice at 6 months of age and scanned using the μCT40, e beam system (Scanco Medical AG, Brüttisellen, Switzerland).

### Statistical Analysis

Differences in bone parameters among different genotypes were assessed by one-way ANOVA. Analysis was performed with JMP Statistical Discovery software (SAS Institute, Inc., Cary, NC). Mean values were calculated along with the standard error of the mean.

## Results

### Absence of preBCR Components Leads to Severe Reductions in Postnatal Skeletal Growth

In the course of our studies of early B cell development, we noticed that when we extracted bone marrow from both male and female λ5^−/−^ mice at 12 weeks of age, their bones appeared more fragile than wild type, irrespective of gender (data not shown). This led us to test whether the preBCR was involved in bone homeostasis.

We gathered a panel of C57BL/6 mice with gene-targeted defects in preBCR formation or function. CD19 is a critical accessory signal transduction partner for the preBCR. Signaling through the preBCR is thus impaired in mice deficient in CD19 (23). These mice have normal numbers of bone marrow proB, preB, immature B, and mature B cells (20, 24, 25). Mice lacking the transmembrane domain of μHC cannot express membrane-bound μHC (19). While they express SLC, they cannot form a functional preBCR or a mature membrane bound IgM B cell receptor (BCR) due to the absence of membrane bound μHC. Absence of both preBCR and BCR results in the total absence of B lineage cells past the early preB cell stage. Mice lacking λ5 cannot form SLC and thus cannot form a preBCR. Loss of SLC leads to a severe reduction in late preB cell numbers. With time, however, uncommon Ig HC-expressing preB cells that managed to evade the preBCR checkpoint and develop into mature B cells accumulate in the BM and the periphery. At 6 months of age, the absolute number of these mature B cells is indistinguishable from wild-type mice (18).

**Figure 1:**
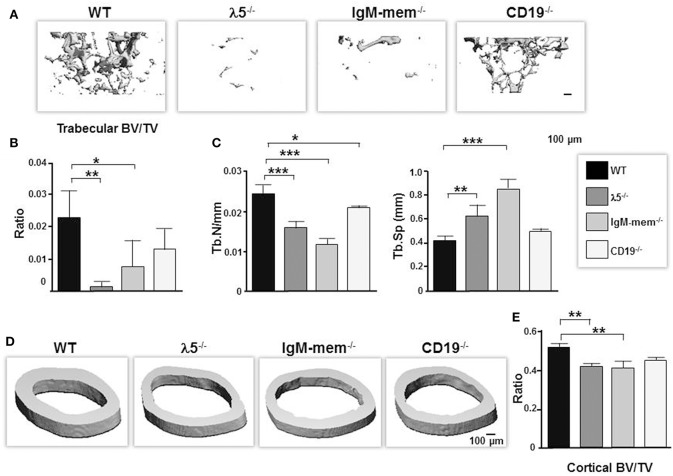
Deficiencies in preBCR formation and functional activity result in loss of cortical and trabecular bone mass. Representative μCT images of femurs from the indicated 6-month old WT and homozygous λ5^−/−^, IgM-mem^−/−^, and CD19^−/−^ C57BL/6 mice. **(A)** Representative 3D reconstructions of trabecular bone beneath the growth plate are shown. Data from 3 mice of each genotype were used to determine the average ratio of **(B)** bone volume to tissue volume (BV/TV) and **(C)** the indicated parameters of trabecular bone. BV, bone volume; TV, total volume; Tb.N, trabecular number; Tb.Sp, trabecular space. **(D)** Representative 3D reconstructions of cortical bone taken at the mid-diaphysis are shown. **(E)** Average bone volume per total volume of cortical bone (BV/TV). Statistical significance was calculated by ANOVA. ^*^p < 0.05, ^**^p < 0.01,^***^p < 0.001. Only comparisons achieving statistical significance in comparison to WT are shown. Scale bar: 100 μm.

At birth, all three mutants had no apparent differences in their skeletal phenotype compared to wild-type controls. However, when we performed 3D μCT imaging of femurs from our panel of 6 month-old mice, we found that impairing preBCR formation or function had led to significant reductions in the quantity of trabecular bone (Figure 1A). CD19^−/−^ mice showed a significant decrease in bone volume (BV; 39% ± 0.01), no significant change in total volume (TV; 4% ± 0.1), and a decrease in BV/TV ratio (43% ± 0.006). IgM-mem^−/−^ mice exhibited decreased bone volume (BV; 63% ± 0.01), increased total tissue volume (TV; 25% ± 0.3), and a decreased BV/TV ratio (68% ± 0.008). λ5^−/−^ mice demonstrated decreased bone volume (BV; 89% ± 0.003), increased total volume (TV; 20% ± 0.06), and a decreased BV/TV ratio (91% ± 0.002) (Figures 1A,B). CD19^−/−^, IgM-mem^−/−^, and λ5^−/−^ mice exhibited a 15, 50, and 35% decrease in trabecular number (Tb.N), respectively (Figure 1C). This resulted in a 18% increase in CD19^−/−^, a 105% increase in IgM-mem^−/−^, and a 50% increase in trabecular space (Tb.Sp) in λ5^−/−^ mice (Figure 1C). Impaired preBCR formation or signaling also resulted in a significant decrease in cortical bone mass (Figure 1D). In comparison to WT controls, the ratio of cortical bone volume to total volume (BV/TV) showed a decrease of 2% ± 0.01 in CD19^−/−^, 10% ± 0.03 in IgM-mem^−/−^, and 8% ± 0.01 in λ5^−/−^ mice (Figure 1E). Together, these data demonstrate that reduced signaling function or loss of preBCR formation in bone marrow impairs maintenance of a normal postnatal skeleton.

λ5^−/−^ mice have normal numbers of mature, recirculating B cells in the bone marrow, and these cells express intact IgM B cell receptors with an intact CD19 accessory signal. Thus, the skeletal abnormality in these mice suggested that the preBCR itself might be playing a significant role in bone homeostasis.

### Deficiencies of preBCR Components Variably Disrupt Postnatal Bone Acquisition and Remodeling

**Figure 2:**
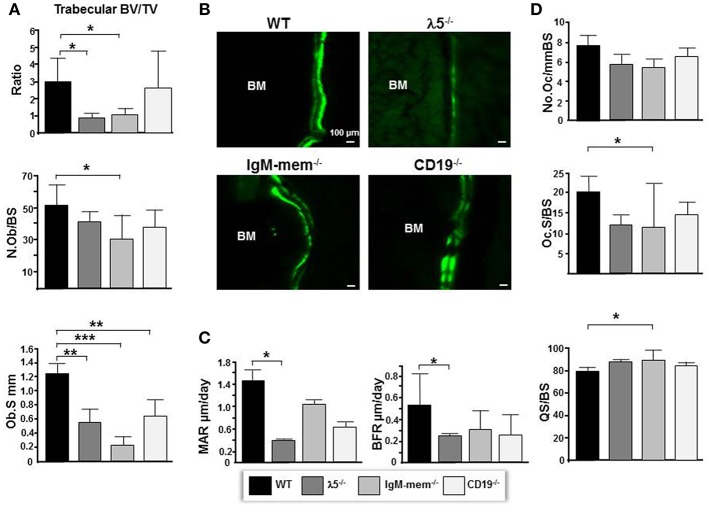
Deficiencies in preBCR formation and function result in decreased osteoblast numbers and bone synthesis. **(A)** Average histomorphometric values pooled from *n* = 3 animals are presented in the graph. Bone parameters shown are trabecular BV/TV ratio, the average number of osteoblasts per bone surface (N.ob/BS), and the average osteoblast surface (Ob.S) for the indicated genotype. **(B)** Undecalcified hindlimbs were embedded in plastic and sectioned laterally. Representative phase and FITC fluorescent images taken at the same exposure time show areas of double labeled bone surfaces in WT and mutant femurs. **(C)** Dynamic histomorphometry was performed on calcein labeled bones. Shown are the average mineralizing parameters for the mineral apposition rate (MAR) and bone formation rate (BFR). **(D)** Frontal sections of femurs were stained for TRAP activity. Shown are the average number of osteoclasts per bone surface (N.Oc/BS), the average osteoclast surface per bone surface (Oc.S/BS), and the quiescent surface to bone surface (QS/BS). ^*^p < 0.05, ^**^p < 0.01,^***^p < 0.001. Only comparisons achieving statistical significance in comparison to WT are shown.

By 6 months of age, normal, healthy, wild-type mice achieve skeletal maturity, and show osteoclast-mediated skeletal remodeling (26, 27). To assess whether a fully functional preBCR influences osteoblast and osteoclast function during skeletal remodeling, we performed histomorphometric analysis. The CD19^−/−^ mice showed no significant change in total volume or bone volume. IgM-mem^−/−^ mice exhibited a significant decrease in bone volume (BV; 63 ± 0.004%), an increase in total volume (TV; 16% ± 0.1), and a decrease in BV/TV ratio (68% ± 0.4). λ5^−/−^ mice also showed a significant decrease in bone volume (BV; 66% ± 0.002), an increase in total volume (TV; 12% ± 0.2), and a significant decrease in the BV/TV ratio (70% ± 0.2) (Figure 2A). These histomorphometric observations were consistent with the μCT findings (Figure 1).

**Figure 3:**
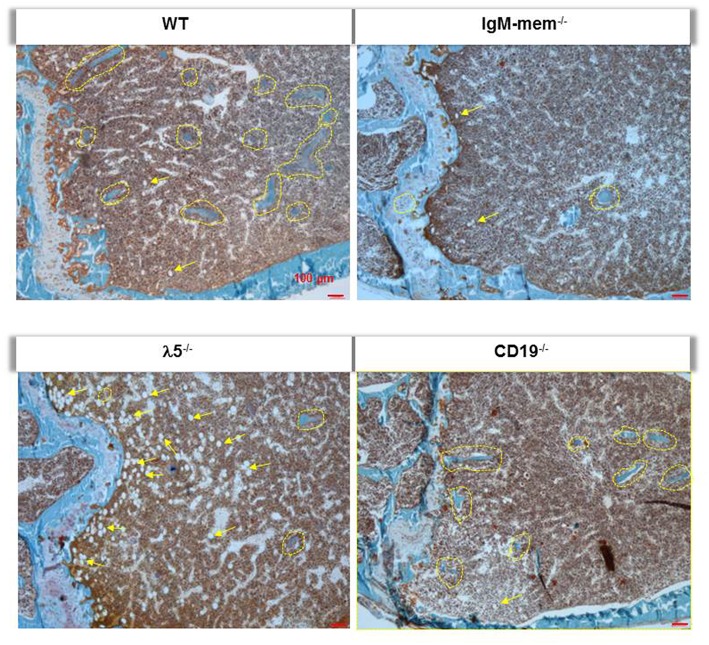
Deficiency of λ5 is associated with decreased adult bone mass. Undecalcified femurs from 6-month-old female WT and the indicated mutant mice were embedded in plastic, sectioned laterally, and stained with Goldner's trichrome. Representative images of the central region surrounding the growth plate are shown at 10x magnification. Individual mineralized trabecular bone beneath the growth plate are identified with yellow dotted lines. Marrow adipocytes in all histologic sections are indicated by yellow arrows.

Goldner's trichrome staining showed an overall decrease in the mineralized matrix beneath the growth plate in IgM-mem^−/−^ and λ5^−/−^ mice, identified with yellow dotted lines (Figure 3). A severe loss of trabecular bone in λ5^−/−^ mice was associated with an increase in marrow adiposity, identified with yellow arrows (Figure 3).

The number of osteoblasts per bone surface (No.Ob/BS) was decreased by 26% ± 11, 40% ± 14, 19% ± 6, and in CD19^−/−^, IgM-mem^−/−^, and λ5^−/−^ mice, respectively (Figure 2A). The osteoblast perimeter (Ob.S) was decreased in CD19^−/−^, IgM-mem^−/−^, and λ5^−/−^, mice by 47% ± 1.4, 81% ± 0.1, and 56% ± 0.21, respectively.

To assess if the decrease in osteoblast size reflects altered osteoblast function in mutant mice, double calcein labeling was performed (Figure 2B). Calcein incorporation was apparent on 30% ± 12.2 and 39% ± 16.6 of the bone surface in CD19^−/−^ and WT mice, respectively; whereas only one quarter of the bone surface in homozygous IgM-mem^−/−^ and λ5^−/−^ femurs was labeled, respectively. In both cortical and trabecular bone surfaces, the calcein signal was noticeably weak in both IgM-mem^−/−^ and λ5^−/−^ mice (Figure 2B). When compared to WT, analysis of the double-labeled bone surfaces revealed a 55% ± 0.6 decrease in the MAR and 46% ± 0.2 decrease in the BFR in the femurs of the CD19^−/−^ mice; a 27% ± 0.5 decrease in the MAR and a 61% ± 0.2 decrease in BFR in the IgM-mem^−/−^ mice (Figure 2C); and a significant decrease of 72% ± 0.04, in the MAR, resulting in a 57% ± 0.2, decrease in BFR of the λ5^−/−^ mice (Figure 2C).

**Figure 4:**
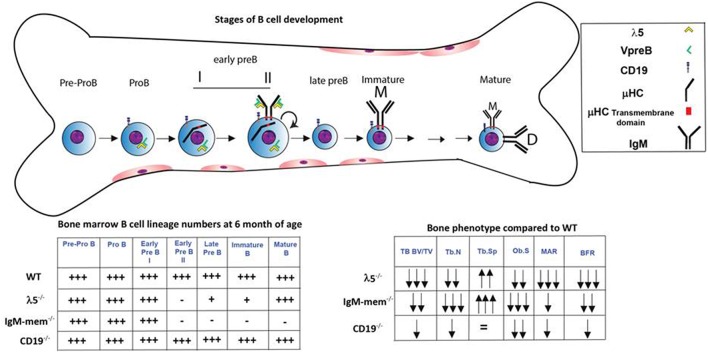
Markers and stages of B cell development that are associated with impairment in postnatal bone mass. B cell development begins in the bone marrow and then continues in the periphery. The stages at which development is affected in the λ5^−/−^, IgM-mem^−/−^, and CD19^−/−^ mice and the relative number of bone marrow B lineage cells at 6 months of age at each stage of development are illustrated (18–20, 24, 25, 28). The two charts summarize the changes in bone marrow B lineage cell numbers and adult bone mass for each genotype.

We next assessed osteoclasts to clarify if the decrease in postnatal bone mass in the mutant mice also reflected increased bone resorption (Figure 2D). We observed that the number of tartrate-resistant acid phosphatase (TRAP)-positive cells (No.Oc) was slightly decreased in all the mutant strains. The osteoclast surface to bone surface ratio (Oc.S/BS) was decreased by 28% ± 3, 44% ± 11, and 41% ± 2 in the CD19^−/−^, IgM-mem^−/−^, and λ5^−/−^ mice (Figure 2D). Similarly, while the erosion surface was decreased by 30% ± 0.3, 60% ± 0.2, and 35% ± 0.1 in the femurs of the CD19^−/−^, IgM-mem^−/−^, and λ5^−/−^ mice, respectively; an increase of 7% ± 1.7, 11% ± 0.2, and 10% ± 0.4 in quiescent surface (QS/BS) was noted. Thus, both osteoblast and osteoclast activity in adult mice is linked to the integrity of preBCR formation and function. Our panel of CD19^−/−^, IgM mem^−/−^, and λ5^−/−^ mice affects a different stage of B lineage development and preBCR and BCR signaling. A comparative summary of impairments in B cell development and the resulting bone defects among our panel of WT and mutant mice is presented in Figure 4.

## Discussion

Both B cells and bone cells develop in the bone marrow, where bone cells are located in the adjacent endosteum. Previous studies have shown that B lineage cells can play an important role in normal bone homeostasis (29). For example, B cells are critical for proper bone repair (30) and there is increased bone loss when B cells are absent (31). Conversely, the stromal cells in the bone are important for normal B cell development (32). This interplay between bone and B cells has been shown to be affected by age (14), hormonal status (14), and disease (33–35). Optimum bone mass depends on a reciprocal balance between bone formation by osteoblasts and bone resorption by osteoclasts. Osteoblasts secrete IL-7, which promotes B cell development (36). Conversely, mature B cells express RANK ligand (RANKL) and osteoprotegrin (OPG), which regulate osteoclast maturation and bone remodeling (37).

Based upon our original unpublished observation of excessively fragile bones in λ5^−/−^ mice, we sought to test the role of the integrity of the preBCR complex on postnatal bone mass. We analyzed postnatal bone in our panel of mutant mice at 6 months of age, when skeletal development is complete (26, 27).

When compared to WT, loss of CD19, which impairs signaling through both the preBCR (23) and BCR but with no effect on numbers of bone marrow proB, preB, immature B, and mature B cells (20, 24, 25), led to a statistically significant decrease in trabecular numbers. However, although the BV/TV ratio, the mineral apposition and rate of bone formation were diminished, the decrease did not achieve statistical significance, and osteoblast surface area in CD19^−/−^ mice was indistinguishable from WT. Thus, while the decrease in trabecular numbers, showing that diminished signaling through the preBCR and/or BCR affected bone; the end effect of the loss of CD19 on bone could be characterized as “mild.”

The effect on bone in mice that lack membrane bound μH chains (IgM mem^−/−^) was more severe. Compared to WT, these mice demonstrated a significant loss of cortical bone in addition to trabecular bone. In these mice, complete loss of the preBCR, the BCR, late preB cells, immature B cells, and mature B cells led to a significant decrease in mineral apposition and the rate of bone formation; as well as a decrease in the number and size of osteoblasts. Given the extent of the developmental block, study of these mice could not determine whether it was the absence of preBCR or BCR signaling, or the absence of late preB cells, immature B cells, and mature B cells that lay behind the altered bone phenotype.

In λ5^−/−^ mice, the BCR signaling apparatus, including membrane-bound IgM and CD19, are intact, and the bone marrow contains normal numbers of mature, recirculating B cells, at 6 month of age, but is deficient in late preB and immature B cells. However, even though these mice have normal number of mature B cells with intact BCR, the defect in bone development matched or exceeded the defects observed in IgM mem^−/−^ mice. λ5^−/−^ mice showed a combination of a decrease in trabecular number and an increase in trabecular bone spacing. λ5^−/−^ bone exhibited decreased postnatal bone acquisition and increased marrow adiposity. The decrease in postnatal bone acquisition in λ5^−/−^ appears to reflect impaired osteoblast activity. A key effect of the loss of λ5 was a decrease in the MAR and the BFR that was more severe than that observed in IgM-mem^−/−^ and CD19^−/−^ mice.

The binding of RANK to RANKL induces osteoclastogenesis and bone resorption. Inflammation can cause B cells to produce higher amounts of RANKL, leading to an increased RANKL/OPG ratio, which drives osteoporosis and bone loss in RA and HIV patients (38). Secretion of OPG, a decoy receptor for RANKL, prevents osteoclastogenesis. Mature B cells express both OPG and RANKL (29). Our findings of decreased bone mass in C57BL/6 IgM-mem^−/−^ mice are consistent with a previous report (29). Bone loss in these B deficient mice was attributed to decreased OPG expression (29), which would be expected to result in increased osteoclastogenesis. However, our studies did not show evidence of increased osteoclastogenesis in IgM-mem^−/−^ mice. The difference in the age of the analyzed mice, 16 weeks for Li et al. and 26 weeks for ours, may account for this variability.

Our studies demonstrate that impaired preBCR formation and decreased preBCR signaling led to a reduction in postnatal bone acquisition and mass, even in the presence of normal number of mature B cells with intact BCR signaling. Moreover, the severity of the bone phenotype is greater when the preBCR is absent (IgM mem^−/−^ and λ5^−/−^ mice) than when its signaling potential is diminished (CD19^−/−^ mice). Unlike plasma cells and mature B cells, precursor B cells secrete very little OPG or RANKL (29, 39). Thus, the decrease in bone mass seen in the preB cell- and preBCR-deficient mice likely reflects one or more mechanisms outside the OPG and RANKL axis.

In summary, loss of the preBCR, with its attendant reduction in late preB cells and immature B cells only, as seen in λ5^−/−^ mice, leads to the same or even slightly more severe bone phenotype than that seen in mice that lack both preBCR and BCR and all B lineage cells past the early preB cell stage. The severity of the phenotype regardless of the integrity of the BCR and the normal numbers of mature B cells suggests a role for the preBCR and/or adequate preB or immature B cell numbers in the acquisition of postnatal bone mass. The fact that trabecular bone formation is reduced when preBCR signaling is reduced emphasizes the importance of a functioning preBCR even when preB and immature B cells are normal.

Our studies suggest a role for the preBCR in maintaining adult bone mass. Among the known ligands for the preBCR are galectin-1 (40), galectin-3 (41, 42), cadherin-17 (43), and heparan sulfate (44). Whether one or more of these ligands, or other unknown factors, are involved in signaling crosstalk between developing preB cells, osteoblasts, and osteoclasts is under active investigation in our laboratories.

## Data Availability

All datasets generated for this study are included in the manuscript.

## Author Contributions

MK, AJ, and HS: study design. MK, HR, and AJ: study conduct and data collection. MK, HR, AJ, and HS: data analysis and data interpretation. MK, PB, SB, AJ, and HS: drafting manuscript. HS: takes responsibility for the integrity of the data analysis.

### Conflict of Interest Statement

The authors declare that the research was conducted in the absence of any commercial or financial relationships that could be construed as a potential conflict of interest.
